# SSR-based evaluation of genetic diversity in populations of Agriophyllum squarrosum L. and Agriophyllum minus Fisch. & Mey. collected in South-East Kazakhstan

**DOI:** 10.18699/VJ20.664

**Published:** 2020-11

**Authors:** Y. Genievskaya, D. Karelova, S. Abugalieva, P. Zhao, G. Chen, Y. Turuspekov

**Affiliations:** Laboratory of molecular genetics, Institute of Plant Biology and Biotechnology, Almaty, Kazakhstan; Laboratory of molecular genetics, Institute of Plant Biology and Biotechnology, Almaty, Kazakhstan; Laboratory of molecular genetics, Institute of Plant Biology and Biotechnology, Almaty, Kazakhstan Department of biodiversity and bioresources, al-Farabi Kazakh National University, Almaty, Kazakhstan; Key laboratory of stress physiology and ecology in cold and arid regions, Northwest Institute of Eco-Environment and Resources, Gansu, China; Key laboratory of stress physiology and ecology in cold and arid regions, Northwest Institute of Eco-Environment and Resources, Gansu, China; Laboratory of molecular genetics, Institute of Plant Biology and Biotechnology, Almaty, Kazakhstan Department of biodiversity and bioresources, al-Farabi Kazakh National University, Almaty, Kazakhstan

**Keywords:** sand rice, Agriophyllum squarrosum, Agriophyllum minus, SSR markers, genetic diversity, population structure, кумарчик песчаный, кумарчик малый, Agriophyllum squarrosum, Agriophyllum minus, SSR-маркеры, генетическое разнообразие, структура популяции

## Abstract

The development of informative polymorphic DNA markers for poorly studied genera is an important step in population analyses of living organisms, including those that play very important ecological roles in harsh environments, such as desert and semi-desert area. Examples of those poorly studied desert species are Agriophyllum squarrosum L. and Agriophyllum minus Fisch. & Mey. However, a recent RNA-sequencing project in A. squarrosum has proposed a large set of hypothetical SSR (simple sequence repeat) markers. In this work, 11 novel polymorphic SSRs were found due to the screening of 24 randomly selected SSRs for three populations of A. squarrosum and one population of A. minus. The analysis of 11 SSRs revealed 16 polymorphic loci in two Agriophyllum species, 8 polymorphic loci within three populations of A. squarrosum, and 6 polymorphic loci in the population of A. minus. Statistical analyses showed high interspecific, but relatively low intraspecific genetic diversity. The phylogenetic clusterization and population structure analysis have demonstrated a clear segregation of A. minus from A. squarrosum, as well as the separation of population 1 from populations 2 and 3 of A. squarrosum. Thus, we identified the set of novel and informative SSR markers suitable for the study of genetic diversity in Agriophyllum.

## Abstract (ru)

Разработка информативных полиморфных ДНК-маркеров для малоизученных родов – необхо- димое и важное условие для эффективного популяционного анализа живых организмов, в том числе играю- щих важную экологическую роль в суровых климатических условиях, в частности в пустынях и полупустынях. Примерами подобных малоизученных пустынных видов являются Agriophyllum squarrosum L. и Agriophyllum minus Fisch. & Mey. В недавнем проекте по РНК-секвенированию A. squarrosum было найдено большое коли- чество гипотетических SSR (простые повторяющиеся последовательности) маркеров. В настоящей работе в результате скрининга 24 случайно отобранных SSR-маркеров для трех популяций A. squarrosum и одной популяции A. minus обнаружены 11 новых полиморфных SSR-маркеров. Изучение этих 11 SSR-маркеров по- казало наличие 16 полиморфных локусов для двух видов Agriophyllum, 8 полиморфных локусов среди трех популяций A. squarrosum и 6 полиморфных локусов для популяции A. minus. Статистический анализ выявил высокое межвидовое, но относительно низкое внутривидовое генетическое разнообразие. Филогенетиче- ский анализ и анализ структуры популяций позволили обнаружить явное разделение на уровне видов, а также дивергенцию популяции 1 A. squarrosum относительно популяций 2 и 3 данного вида. Таким образом, нами идентифицирован набор новых и информативных SSR-маркеров, подходящих для изучения генетиче- ского разнообразия Agriophyllum.

## Introduction

Xerophytes and psammophytes (plants with adaptations to survive in water-deficiency environment) become the main plants in the desert region. Despite the harsh environmental conditions, many of these plants have adapted and became a part of complex and diverse desert ecosystems. Due to the wide distribution of desert territories, the study of its wild flora is very important for the ecological prediction and conservation of biodiversity. The Central Asia region, including Kazakhstan, is having numerous large and small sand deserts, such as Gobi, Taklamakan, Karakum, Kyzylkum and Moiynkym. Deserts and semi-deserts occupy more than half of Kazakhstan territory and keep growing (Issanova et al., 2015). The major representatives of wild desert flora here are herbaceous plants, subshrubs, shrubs and subtrees. The list of dominating species includes genera Artemisia, Salsola, Ferula, Arthrophytum, Calligonum, Ammodendron, Haloxylon, and Agriophyllum (Rachkovskaya et al., 2003). Many of them are endemic for the Central Asian and Kazakhstan deserts.

Agriophyllum squarrosum L. and Agriophyllum minus Fisch. & Mey. are important desert species in Kazakhstan. They belong to the tribe Corispermeae within the subfamily Chenopodioideae of the family Chenopodiaceae (Kühn, 1993). The genus Agriophyllum includes five species, and four of them, including A. squarrosum, grow in Kazakhstan (Ageeva et al., 1960). A. squarrosum is also widely spread in all Central Asia territory, Caucasus, and China. Local people in the sandy desert regions of China consume the seed of the species during periods of food shortage, and refer to the plant as ‘shami’ in Chinese, which translates as ‘sand rice’ (Chen et al., 2014). Sand rice is an example of psammophyte perfectly adapted for harsh desert environmental conditions. Morphologically it is a shrub-like plant with a height ranging from 20 to 100 cm. The stem of sand rice at the young plant stage is firm, branched, green, and covered with short hairs. Leaves are small, sessile, green, and usually linear. Small flowers are organized in a spike-like inflorescence. A. squarrosum blossoms in the late summer and early fall, after that ovoid shape seeds are formed. Seeds of sand rice are very light and covered by a thin husk. After ripening, the husk is cracked into two parts, and seeds are easily dispersed by the wind. The root system is represented by a long taproot penetrating deep into the soil to access stored moisture, and almost equally long lateral roots branching near the soil surface and helping to fix plant in loose sand. Although, there is a lack of information related to the structure of the sand rice’s genome, the transcriptomic analysis of A. squarrosum (2n = 18) had showed presence of 67 741 unigenes and approximately 43 % of them were annotated (Zhao et al., 2014).

Throughout history, sand rice was used for diverse purposes. Aboveground organs (stem and leaves) are eaten by both wild animals and livestock of farmers in arid and semi-arid regions, especially in Western Kazakhstan on camel pasture. Historically, nutritious seeds of sand rice were an alternative to cereals not surviving in hot deserts. In China and Mongolia, the local villagers consume sand rice seed in a variety of dishes. There are many reports about the rich nutrition value of A. squarrosum seeds close to its widely-used as food quinoa seeds (Chen et al., 2014). However, sand rice is not a domesticated plant species with several agriculturally unfavorable traits, such as fragile spikes and light seeds. Still, the works on possible domestication of sand rice as a novel crop are reported (Chen et al., 2014). In addition to nutrition purposes, sand rice had found its application in medicine. Back in the days, it was used as antipyretic and analgesic medicine (Gong et al., 2012; Chen et al., 2014). It was reported that the extract of A. squarrosum decreases blood glucose levels in type 2 diabetic mice and has the potential for further medical researches (Saqier et al., 2019). Sand rice is useful in combat against shifting sands (Wen-Ming et al., 2004). Climate change and human activities led to the growth of sand desert areas and the migration of sand dunes to agricultural territories. The structure of the sand rice root system and its ability to form seed banks in active sand dunes allow the fixation of the sand surrounding the plant. Thus, A. squarrosum has a tremendous potential together with other psammophytes to be used in a large-scale sand fixation (Liu et al., 2007; Ma, Liu, 2008). This species is an interesting model for different studies of morphology and physiology of desert plants. For example, earlier A. squarrosum was already used for the study of growth under drought conditions (Mo et al., 1997; Huang et al., 2008) and for the study of fertilizer effect on psammophytes under different rainfall conditions (Yuan et al., 2019).

Endemic species with great economic potential like sand rice are an interesting subject for genetic and molecular researches. One of the most common methods utilized for the studies on biodiversity conservation, population and phylogenetic studies of wild plant species is the usage of molecular DNA markers (Nybom, 2004). Examples of successful application of the most common DNA markers in plants include random amplification of polymorphic DNA (RAPD) (Nybom, Bartish, 2000), amplified fragment-length polymorphisms (AFLP) (Zhang C. et al., 2018), and other nuclear and chloroplast DNA markers (Abugalieva et al., 2017; Almerekova et al., 2018; Turuspekov et al., 2018). Nuclear ribosomal internal transcribed spacer (nrITS) region and five chloroplast DNA (cpDNA) fragments have been used earlier for the study of population dynamics of A. squarrosum in China (Qian et al., 2016). The maturase K (matK ) gene of the chloroplast genome and nrITS were used for comparison of A. squarrosum and A. minus populations in two regions of Kazakhstan (Genievskaya et al., 2017). The literature survey suggests that there is a limited information on the study of Agriophyllum species by using SSR markers. However, a recent RNA-sequencing project of A. squarrosum populations in China has suggested several thousands of potential SSR markers for this species (Zhang J. et al., 2018).

In this study, we selected 24 SSR markers from this Agriophyllum genome resequencing project and used them for the assessment of genetic diversity within and among populations of A. squarrosum, and between A. squarrosum and A. minus.

## Materials and methods

**Fig. 1.**
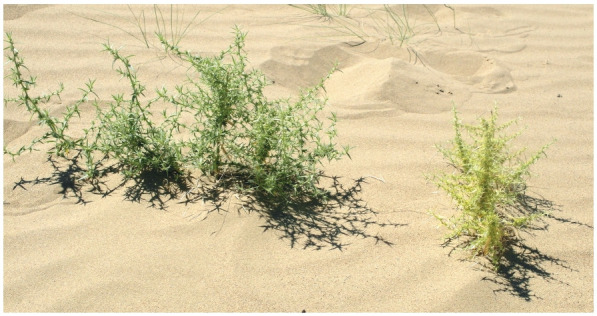
A. squarrosum (on the left) and A. minus (on the right) in Moyynkum desert.

**Plant material.** In total, leaf samples of four wild Agriophyllum populations were collected in South-East Kazakhstan and used for the analysis (Table 1). The list included three populations of A. squarrosum and one population of A. minus sampled in Moyynkum desert of Almaty region in South-East Kazakhstan (Fig. 1). Population 1 of A. squarrosum was collected in 2016, while the populations 2 and 3 of A. squarrosum and population 1 of A. minus were collected in 2019. The distances between populations were at least four kilometers, and plants within the population were sampled in at least 50 meters apart.

**Table 1.**
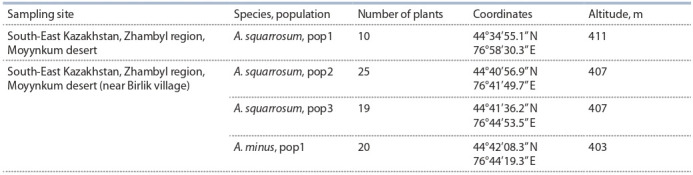
Geographical locations of sampling sites

**DNA extraction, amplification and SSR marker assessment.** Five young leaves from each sample were dried in silica gel. The total genomic DNA was extracted from dry leaf tissues using CTAB method (Doyle, 1991). The quality and concentration of extracted DNA were assessed via spectrophotometric test, and 1 % agarose gel electrophoresis.

(Supplementary Table)^1^ were selected from 6150 SSRs in the sequence of A. squarrosum genome reported by Zhang J. and co-authors (2018). SSR motives and expected sized of alleles were obtained from the same source.

^1^ Supplementary Table is available in the online version of the paper: http://www.bionet.nsc.ru/vogis/download/pict-2020-24/appx10.pdf



Annealing temperature (T_a_ ) for primer pairs and concentration of reagents in a PCR reaction mix were determined empirically. All successful PCR reactions were performed in total 16 μl volumes, including 4 mM of each dNTP, 2 mM of MgCl_2_, 6.4 mM of primer mix (forward + reverse), 1.6 U of Taq polymerase and 50 ng of DNA. The amplification was performed in Veriti Thermocycler (Applied Biosystems, Foster City, CA, USA) with an initial denaturation step at 94 °C for 3 minutes, followed by 40 cycles of 94 °C for 30 seconds, optimized T_a_ °C (see Suppl. Table) for 45 seconds and 72 °C for 1.5 minutes. The final extension step was at 72 °C for 10 minutes. The PCR products were separated on 6 % polyacrylamide gel (PAG). The SSR profile image was captured using the GelDoc gel documentation unit (Bio-Rad Laboratories, Hercules, CA, USA). Allele sizes were estimated visually based on the size of ladder bands and the control sample on each gel.

Statistical analysis. Nei’s genetic diversity indices were calculated via POPGENE software ver. 1.32 (Yeh et al., 1997); the polymorphism information content (PIC) (Botstein et al., 1980) was calculated as follows: PIC = Σ(1 – p^2^_i_ ), where pi is the frequency of the i-th band or percentage of individuals in which the fragment is present. Principal coordinates analysis (PCoA) and unweighted pair group method with arithmetic mean (UPGMA) hierarchical clustering were performed based on genetic distances among populations and species. PCoA plot was made via GenAlEx (Genetic Analysis in Excel) ver. 6.5 software (Peakall, Smouse, 2012), UPGMA analysis was performed in R statistical software environment (R Core Team, 2018). Bayesian clustering was based on the use of the STRUCTURE software ver. 2.3.4 (Pritchard et al., 2000). The value of K was set from 1 to 5 with five iterations for each value of K. Both, length of burn-in period, and the number of Markov Chain Monte Carlo (MCMC) repeat after burn-in was set at 100 000. Web-tool STRUCTURE HARVESTER (Earl, von Holdt, 2011) based on Evanno’s method (Evanno et al., 2005) was used to determine the best fit value of K. The molecular variance (AMOVA) test was calculated using GenAlEx software.

## Results

**Performance of SSR markers in Agriophyllum species**

Initially, 24 SSR markers were chosen for the analysis (see Suppl. Table), however, successful amplification was performed for 18 markers only. Five of 18 SSRs were multilocus markers, while other 13 SSRs were single-locus. In total, 18 markers allowed identification of 23 loci (Table 2), of which 16 were polymorphic and suitable for the analysis (Table 3).

**Table 2.**
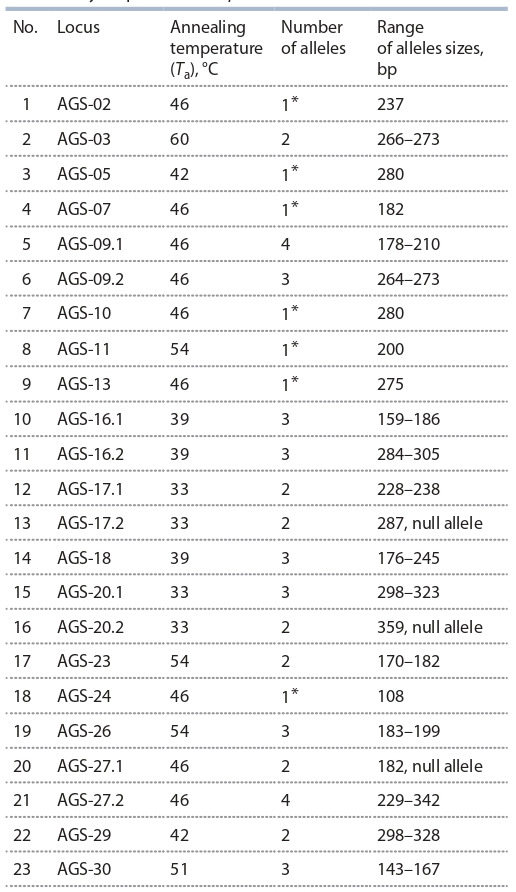
The characteristics of SSR loci successfully amplified in A. squarrosum and A. minus * Monomorphic loci excluded from further analysis.

**Table 3.**
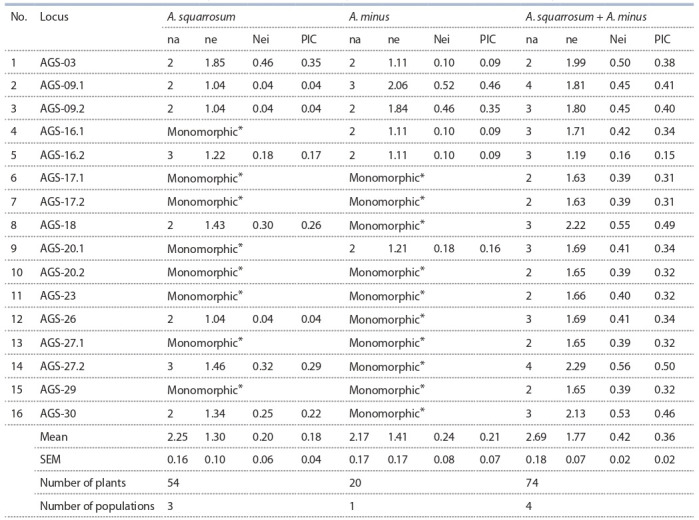
Assessment of genetic diversity in populations of Agriophyllum species based on SSR marker analysis Note. na – number of alleles per locus; ne – number of effective alleles; Nei – Nei’s genetic diversity index; PIC – polymorphism information content; SEM – standard error of mean. * Monomorphic loci were not considered for the calculation of mean and SEM values.

The screening of sixteen SSR loci has allowed the identification of 43 alleles in the analysis of three populations of A. squarrosum and one population of A. minus. The molecular sizes of alleles in loci were ranged from 143 to 342 bp. The number of alleles in the study of all four populations varied from 2 to 4.

In total, eight polymorphic loci were observed in A. squarrosum, six polymorphic loci were found in A. minus, and 16 loci had demonstrated polymorphism between two species (see Table 3). In total, 20 alleles were identified exclusively in A. squarrosum, 16 alleles were exclusive for A. minus, and 7 alleles were found in both species. The average number of alleles per locus in polymorphic loci was 2.69 ± 0.70 for both species, 2.25 ± 0.16 for A. squarrosum, and 2.17 ± 0.17 for A. minus, respectively.

**Fig. 2.**
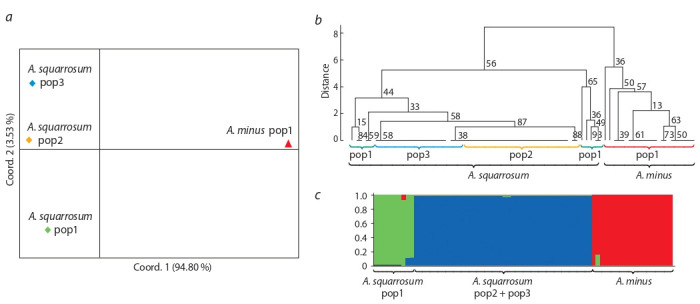
Population structure among studied Agriophyllum species. a, PCoA of three A. squarrosum and one A. minus populations; b, UPGMA dendrogram showing clusters among 74 samples of both species; c, Bayesian clustering of 74 Agriophyllum samples at K = 3.

Nei’s index and PIC value were calculated separately for polymorphic SSR loci of A. squarrosum, A. minus, and jointly for two Agriophyllum species (see Table 3). The genetic distance-based PCoA plot suggested a clear separation of A. minus from populations of A. squarrosum, as well as distinguishing of the population 1 from populations 2 and 3 in A. squarrosum (Fig. 2, a). The UPGMA tree had also demonstrated two clusters corresponding to A. squarrosum and A. minus. The portion of samples in population 1 of A. squarrosum was clustered together with populations 2 and 3, while the remaining part formed a separate subcluster (see Fig. 2, b). Bayesian distance-based analysis of the structure among two Agriophyllum species was congruent with the UPGMA clusterization and also indicated that K = 3 is an optimal number of groups in the study (see Fig. 2, c).

AMOVA results for the set of data containing all Agriophyllum samples revealed that the majority (88 %) of the genetic diversity in Agriophyllum exists between two species rather than within them (Table 4).

**Table 4.**
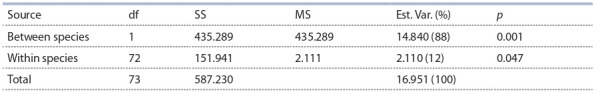
AMOVA in two species of Agriophyllum Note. df – degree of freedom; SS – sum of squares; MS – mean squares; Est. Var. – estimated variance; p – significance level.

**Genetic diversity in A. squarrosum populations**

Part of this study was focused on the assessment of the genetic diversity within and between populations of A. squarossum. In total, 26 alleles were identified in eight SSR loci. The largest number of unique alleles was found in population 1 (8 alleles), while population 2 had only one unique allele (Table 5). The largest values of Nei’s index (0.27 ± 0.16) and PIC value (0.25 ± 0.09) were observed in population 1.

**Table 5.**
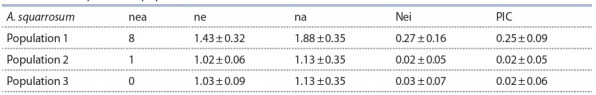
Average values of diversity statistics for eight polymorphic SSR loci across three A. squarrosum populations Note. nea – number of alleles exclusive for population; na – number of alleles per locus; ne – number of effective alleles; Nei – Nei’s genetic diversity index; PIC – polymorphism information content.

The AMOVA for A. squarrosum had shown a higher molecular variance within populations than the variance among populations (Table 6).

**Table 6.**
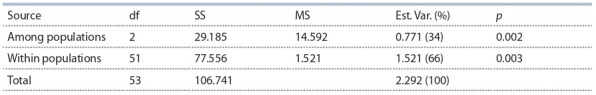
AMOVA among and within three populations of A. squarrosum Note. df – degree of freedom; SS – sum of squares; MS – mean squares; Est. Var. – estimated variance; p – significance level.

## Discussion

The availability of informative DNA markers is advantageous for the study of rare species, their population structure, and for the development of a proper conservation strategy to prevent their extinction (Adams, Turuspekov, 1998; Kramer, Havens, 2009). SSR markers are one of those types of DNA markers that reliably utilized in population structure analysis due to their hyper-variability, co-dominance, and high reproducibility (Aitken et al., 2005). Successful applications of SSR markers for the identification, classification, and taxonomy of Chenopodiaceae species have been reported previously (Borger et al., 2008; Prinz et al., 2009; Sampson, Byrne, 2012; Nachtigall et al., 2016). However, for the genus Agriophyllum, there were no previous studies with the use of SSRs. The situation was changed recently due to the RNAsequencing project of A. squarrosum accessions, and a large number of hypothetical SSR markers were suggested for population studies (Zhang J. et al., 2018). Hence, in this work 24 SSRs were randomly selected for the assessment of three populations of A. squarrosum and one population of A. minus collected in South-East Kazakhstan. The assessment of selected SSR markers resulted in the identification of 11 novel SSR markers that allowed the identification of 16 polymorphic loci in two Agriophyllum species (see Table 3). The large percentage of molecular variance (see Table 4) and a large Table 5. Average values of diversity statistics for eight polymorphic SSR loci across three A. squarrosum populations A. squarrosum nea ne na Nei PIC Population 1 8 1.43 ± 0.32 1.88 ± 0.35 0.27 ± 0.16 0.25 ± 0.09 Population 2 1 1.02 ± 0.06 1.13 ± 0.35 0.02 ± 0.05 0.02 ± 0.05 Population 3 0 1.03 ± 0.09 1.13 ± 0.35 0.03 ± 0.07 0.02 ± 0.06 Note. nea – number of alleles exclusive for population; na – number of alleles per locus; ne – number of effective alleles; Nei – Nei’s genetic diversity index; PIC – polymorphism information content. Table 4. AMOVA in two species of Agriophyllum Source df SS MS Est. Var. (%) p Between species 1 435.289 435.289 14.840 (88) 0.001 Within species 72 151.941 2.111 2.110 (12) 0.047 Total 73 587.230 16.951 (100) Note. df – degree of freedom; SS – sum of squares; MS – mean squares; Est. Var. – estimated variance; p – significance level. genetic distance (see Fig. 2) observed between A. squarrosum and A. minus indicated good discriminative power of studied SSR markers and confirmed a significant genetic difference reported between these species earlier (Genievskaya et al., 2018). Fifteen SSR loci used for both species were in intermediate diversity group with PIC values ranged between 0.32 and 0.50, and Nei’s diversity index ranged between 0.39 and 0.56 (see Table 3).

When species were analyzed separately, 8 out of 16 loci were polymorphic for A. squarrosum, and 6 loci were polymorphic for A. minus (see Table 3). The average diversity in A. minus was slightly higher than in A. squarrosum and lower than the average interspecies diversity (see Table 3). Thus, A. squarrosum and A. minus have maintained a low level of genetic diversity. It has been demonstrated that annuals like Agriophyllum or short-lived perennials usually demonstrate low levels of genetic diversity compared with long-lived and outcrossing species (Austerlitz et al., 2000). However, the limited amount of samples in studied species (particularly in A. minus), their close geographical locations, and a relatively small number of polymorphic SSR markers used for the analysis could influence obtained results.

The study of the genetic diversity within A. squarrosum suggested that 66 % of the total variation was within and 34 % between populations (see Table 6), indicating that the difference of population 1 from populations 2 and 3 was rather substantial. The presence of relatively high diversity within populations may be explained by the geographical proximity of A. squarrosum populations used in this study (see Table 1), which may cause a high rate of gene flow in a limited area (Conner, Hartl, 2004). Populations 2 and 3 of A. squarrosum had demonstrated an extremely low level of genetic diversity in comparison with population 1 (see Table 5), but the fact that samples from each population clustered together using multiple analyses (PCoA, UPGMA, Bayesian clustering) (see Fig. 2) indicates that there is a high level of heterogeneity in the species. The grouping of 5 samples from population 1 on the UPGMA tree and on the STRUCTURE bar plot into separate subcluster in the entire species cluster may indicate the presence of some barriers between groups of samples within population 1 interfering gene flow. Therefore, even if no physical obstructions were found during the sample collection, probably, two groups in population 1 may be considered as subpopulations (Waples, Gaggiotti, 2006). This assumption is also supported by the relatively higher genetic diversity in population 1 comparing with the other two A. squarrosum populations (see Table 5).

## Conclusion

In this study, sequences of 24 pairs of oligonucleotides for SSR markers were randomly selected from A. squarrosum genome resequencing project, and used for the study of genetic diversity in the genus Agriophyllum. Hence, it is the first report exploring the performance of novel SSR markers in the genetic analysis of this genus. The study revealed 16 polymorphic loci in eleven SSR markers using two Agriophyllum species, 8 polymorphic loci within three populations of A. squarrosum, and 6 polymorphic loci within the population of A. minus. Statistical analyses showed high interspecific, but relatively low genetic diversity in populations 2 and 3 of A. squarrosum. The phylogenetic clusterization and analysis and population structure analysis demonstrated clear segregation of A. minus from A. squarrosum, as well as the separation of population 1 of A. squarrosum form populations 2 and 3. As a result, we identified the set of novel and informative SSR markers suitable for the study of genetic diversity in Agriophyllum species. These results provide an important contribution to the population study and approaches for the development of conservation mechanisms for Agriophyllum species.

## Conflict of interest

The authors declare no conflict of interest.

## References

Abugalieva S., Volkova L., Genievskaya Y., Ivaschenko A., Kotukhov
Y., Sakauova G., Turuspekov Y. Taxonomic assessment of
Allium
species from Kazakhstan based on ITS and matK markers.
BMC Plant Biol. 2017;17(S2):258. DOI 10.1186/s12870-017-1194-0.

Adams R.P., Turuspekov Y. Taxonomic reassessment of some Central
Asian and Himalayan scale-leaved taxa of Juniperus (Cupressaceae)
supported by random amplification of polymorphic DNA.
Taxon. 1998;47(1):75-83. DOI 10.2307/1224021.

Ageeva N.T., Baitenov M.B., Goloskokov V.P., Kornilova V.S., Pavlov
N.V., Poljakov P.P. Agriophyllum. In: Pavlov N.V. (Ed.) Flora of
Kazakhstan. Vol. III. Alma-Ata: Academy of Sciences of KazSSR,
1960;241-243. (in Russian)

Aitken K.S., Jackson P.A., McIntyre C.L. A combination of AFLP and
SSR markers provides extensive map coverage and identification of
homo(eo)logous linkage groups in a sugarcane cultivar. Theor. Appl.
Genet. 2005;110:789-801. DOI 10.1007/s00122-004-1813-7.

Almerekova S., Abugalieva S., Mukhitdinov N. Taxonomic assessment
of the Oxytropis species from South-East of Kazakhstan. Vavilovskii
Zhurnal Genetiki i Selektsii = Vavilov Journal of Genetics and
Breeding. 2018;22(2):285-290. DOI 10.18699/VJ18.362.

Austerlitz F., Mariette S., Machon N., Gouyon P.H., Godelle B. Effects
of colonization processes on genetic diversity: differences between
annual plants and tree species. Genetics. 2000;154(3):1309-1321.

Borger C.P.D., Yan G., Scott J.K., Walsh M.J., Powles S.B. Salsola tragus
or S. australis (Chenopodiaceae) in Australia – untangling taxonomic
confusion through molecular and cytological analyses. Aust.
J. Bot. 2008;56(7):600-608. DOI 10.1071/bt08043.

Botstein D., White R.L., Skolnick M., Davis R.W. Construction of a
genetic linkage map in man using restriction fragment length polymorphisms.
Am. J. Hum. Genet. 1980;32:314-331.

Chen G., Zhao J., Zhao X., Zhao P., Duan R., Nevo E., Ma X. A psammophyte
Agriophyllum squarrosum (L.) Moq.: a potential food
crop. Genet. Resour. Crop Evol. 2014;61(3):669-676. DOI 10.1007/
s10722-014-0083-8.

Conner J.K., Hartl D.L. A Primer of Ecological Genetics. Oxford Univ.
Press, 2004.

Doyle J. DNA protocols for plants. In: Molecular Techniques in Taxonomy.
Berlin; Heidelberg: Springer, 1991;283-293.

Earl D.A., von Holdt B.M. Structure Harvester: a website and program
for visualizing STRUCTURE output and implementing the Evanno
method. Conserv. Genet. Resour. 2011;3:429-431. DOI 10.1007/
s12686-011-9548-7.

Evanno G., Regnaut S., Goudet J. Detecting the number of clusters of
individuals using the software STRUCTURE: a simulation study.
Mol. Ecol. 2005;14(8):2611-2620. DOI 10.1111/j.1365-294X.2005.
02553.x.

Genievskaya Y., Abugalieva S., Zhubanysheva A., Turuspekov Y.
Morphological
description and DNA barcoding study of sand rice
(Agriophyllum squarrosum, Chenopodiaceae) collected in Kazakhstan.
BMC Plant Biol. 2017;17(S1):177. DOI 10.1186/s12870-017-
1132-1.

Gong B., Zhan K.X., Zhou Y.H., Zhang L., Hui Y.Q., Li Y.S. Separation
and identification of chemical constituents from Agriophyllum
squarrosum (L.) Moq. Mod. Chinese Med. 2012;14(10):7-11.

Huang Y.X., Zhao X.Y., Zhang H.X., Luo Y.Y., Mao W. Responses of
Agriophyllum squarrosum phenotypic plasticity to the changes of
soil nutrient and moisture contents and population density. J. Appl.
Ecol. 2008;19(12):2593-2598.

Issanova G., Abuduwaili J., Kaldybayev A., Semenov O., Dedova T.
Dust storms in Kazakhstan: frequency and division. J. Geol. Soc.
India. 2015;85(3):348-358. DOI 10.1007/s12594-015-0224-5.

Kramer A.T., Havens K. Plant conservation genetics in a changing
world. Trends Plant Sci. 2009;14(11):599-607. DOI 10.1016/j.
tplants.2009.08.005.

Kühn U. Chenopodiaceae. In: Kubitzki K., Rohwer J.G., Bittrich V.
(Eds.). The Families and Genera of Vascular Plants. II. Flowering
Plants: Dicotyledons, Magnoliid, Hamamelid and Caryophyllid
families. Berlin; Heidelberg; New York: Springer, 1993;253-280.

Liu Z., Yan Q., Liu B., Ma J., Luo Y. Persistent soil seed bank in Agriophyllum
squarrosum (Chenopodiaceae) in a deep sand profile: variation
along a transect of an active sand dune. J. Arid. Environ. 2007;
71(2):236-242. DOI 10.1016/j.jaridenv.2007.03.003.

Ma J., Liu Z. Spatiotemporal pattern of seed bank in the annual psammophyte
Agriophyllum squarrosum Moq. (Chenopodiaceae) on the
active sand dunes of northeastern Inner Mongolia, China. Plant Soil.
2008;311(1-2):97-107. DOI 10.1007/s11104-008-9661-x.

Mo W., Natori T., Jiang S., Nishimura N., Omasa K. Responses of photosynthesis
and water use to drought in two desert annuals, Agriophyllum
squarrosum and Bassia dasyphylla. J. Arid Land Studies.
1997;7:185-195.

Nachtigall M., Bülow L., Schubert J., Frese L. Development of SSR
markers for the genus Patellifolia (Chenopodiaceae). Appl. Plant
Sci. 2016;4(8):1600040. DOI 10.3732/apps.1600040.

Nybom H. Comparison of different nuclear DNA markers for estimating
intraspecific genetic diversity in plants. Mol. Ecol. 2004;13(5):
1143-1155. DOI 10.1111/j.1365-294x.2004.02141.x.

Nybom H., Bartish I.V. Effects of life history traits and sampling strategies
on genetic diversity estimates obtained with RAPD markers in plants. Perspect. Plant Ecol. Evol. Syst. 2000;3(2):93-114. DOI
10.1078/1433-8319-00006.

Peakall R., Smouse P.E. GenAlEx 6.5: genetic analysis in Excel. Population
genetic software for teaching and research – an update. Bioinformatics.
2012;28(19):2537-2539. DOI 10.1093/bioinformatics/
bts460.

Prinz K., Hensen I., Schie S., Debener T., Weising K. Microsatellite
markers for the tetraploid halophyte Suaeda maritima (L.) Dumort.
(Chenopodiaceae) and cross‐species amplification in related taxa.
Mol. Ecol. Resour. 2009;9(4):1247-1249. DOI 10.1111/j.1755-0998.
2009.02620.x.

Pritchard J.K., Stephens M., Donnelly P. Inference of population structure
using multilocus genotype data. Genetics. 2000;155(2):945-959.

Qian C., Yin H., Shi Y., Zhao J., Yin C., Luo W., Dong Z., Chen G.,
Yan X., Wang X.-R., Ma X.-F. Population dynamics of Agriophyllum
squarrosum, a pioneer annual plant endemic to mobile sand dunes,
in response to global climate change. Sci. Rep. 2016;6(1):26613.
DOI 10.1038/srep26613.

Rachkovskaya E.I., Safronova I.N., Volkova E.A. Botanical geography
of Kazakhstan and Middle Asia (desert region). St. Petersburg, 2003;
192-222. (in Russian).

R Core Team. R: a language and environment for statistical computing.
R Foundation for Statistical Computing, Vienna, Austria, 2018. URL
https://www.R-project.org/

Sampson J.F., Byrne M. Genetic diversity and multiple origins of polyploid
Atriplex nummularia Lindl. (Chenopodiaceae). Biol. J. Linn.
Soc. 2012;105(1):218-230. DOI 10.1111/j.1095-8312.2011.01787.x.

Saqier Bao S., Han S., Ao W. Effects of Agriophyllum squarrosum
extracts
on glucose metabolism in KKAy mice and the associated
underlying mechanisms. J. Ethnopharmacol. 2019;241:112009.
DOI 10.1016/j.jep.2019.112009.

Turuspekov Y., Genievskaya Y., Baibulatova A., Zatybekov A., Kotuhov
Y., Ishmuratova M., Imanbayeva A., Abugalieva S. Phylogenetic
taxonomy of Artemisia L. species from Kazakhstan based on
MatK analyses. Proc. Latv. Acad. Sci. B Nat. Exact Appl. Sci. 2018;
72(1):29-37.

Waples R.S., Gaggiotti O. What is a population? An empirical evaluation
of some genetic methods for identifying the number of gene
pools and their degree of connectivity. Mol. Ecol. 2006;15(6):1419-
1439. DOI 10.1111/j.1365-294x.2006.02890.x.

Wen-Ming B., Xue-Mei B., Ling-Hao L. Effects of Agriophyllum
squarrosum seed banks on its colonization in a moving sand dune
in Hunshandake Sand Land of China. J. Arid. Environ. 2004;59(1):
151-157. DOI 10.1016/j.jaridenv.2004.01.006.

Yeh F., Yang R., Boyle T., Ye Z., Mao J. POPGEN Ver. 1.32. The userfriendly
software for population genetic analysis. Alberta, Canada:
Molecular Biology and Biotechnology Center; Univ. of Alberta,
1997.

Yuan M., Xiao H., Wang R., Duan Y., Cao Q. Effects of changes in
precipitation pattern and of seaweed fertilizer addition on plant traits
and biological soil crusts. J. Appl. Phycol. 2019;31(6):3791-3802.
DOI 10.1007/s10811-019-01838-1.

Zhang C., Sun M., Zhang X., Chen S., Nie G., Peng Y., Huang L.,
Ma X. AFLP-based genetic diversity of wild orchardgrass germplasm
collections from Central Asia and Western China, and the
relation to environmental factors. PLoS One. 2018;13(4):e0195273.
DOI 10.1371/journal.pone.0195273.

Zhang J., Zhao P., Zhao J., Chen G. Synteny-based mapping of causal
point mutations relevant to sand rice (Agriophyllum squarrosum)
trichomeless1 mutant by RNA-sequencing. J. Plant Physiol. 2018;
231:86-95. DOI 10.1016/j.jplph.2018.09.003.

Zhao P., Capella-Gutiérrez S., Shi Y., Zhao X., Chen G., Gabaldón T.,
Ma X.-F. Transcriptomic analysis of a psammophyte food crop,
sand rice (Agriophyllum squarrosum) and identification of candidate
genes essential for sand dune adaptation. BMC Genomics. 2014;15:
872. DOI 10.1186/1471-2164-15-872.

